# Low-Coverage Whole-Genome Sequencing (lcWGS) in Cattle: Analysis of Potential and Prospects for Application

**DOI:** 10.3390/ani15243538

**Published:** 2025-12-08

**Authors:** Olga Kostyunina, Nikita Koldichev, Gleb Nemkovskiy, Alexey Traspov, Anton Ermilov, Faridun Bakoev, Dmitriy Chesnokov, Anna Panova, Kseniia Antonovskaia, Alexander Kusnetzov, Vladimir Belyakov

**Affiliations:** 1 LLC «WESTTRADE Ltd.», 115201 Moscow, Russia; koldichevnikita@gmail.com (N.K.); gbnem@mail.ru (G.N.); traspovalex@gmail.com (A.T.); de_lamak@mail.ru (A.E.); bakoevfaridun@yandex.ru (F.B.); chesnokovdmitrii@yandex.ru (D.C.); annak_2000@mail.ru (A.P.); antonovskaya.kn@gmail.com (K.A.); kuz.ab@icloud.com (A.K.); bel.vk@yandex.ru (V.B.); 2 Department of Information Technology and Medical Data Processing, Institute of Digital Biodesign and AI in Medicine, Sechenov First Moscow State Medical University, 119991 Moscow, Russia; 3 Institute of Biomedical Sciences (IBS), Pirogov Moscow State Medical University, 119571 Moscow, Russia

**Keywords:** cattle, low-pass sequencing, lcWGS, imputation

## Abstract

Whole-genome studies in cattle play a key role in exploring both individual and population-level genetic variability. Recently, low-coverage whole-genome sequencing (0.5–2×) has been considered as an alternative to traditional approaches. Low-coverage whole-genome sequencing (lcWGS), which provides uniform coverage of the entire genome at relatively low cost, combined with subsequent imputation, enables the reconstruction of genotypes with high accuracy and density. lcWGS enables detection of rare and functionally important variants and provides exploratory potential for structural variation analysis; however, accurate SV imputation still presents significant challenges. The aim of this review is to analyze the potential and prospects of lcWGS as a tool for genomic selection and genetic studies in cattle. The review systematizes current advances in the application of lcWGS in cattle, focusing on imputation accuracy, factors affecting it, and the comparative efficiency of different software solutions. A literature survey was conducted using PubMed and Google Scholar databases, with preference given to original studies, systematic reviews, and large-scale projects addressing imputation accuracy, reference panel composition and size, cost-effectiveness, and practical applications of lcWGS in cattle genomics. Key factors influencing efficiency include sequencing depth, reference panel size and composition, as well as the choice of imputation algorithm. lcWGS represents a cost-effective and powerful alternative to traditional genome-wide approaches, capable of capturing rare and breed-specific variants; however, its application to structural variation still requires methodological improvement and integration with high-resolution reference pangenomes or long-read sequencing. Despite significant progress and the high potential of lcWGS in cattle genomics, several challenges and limitations remain, requiring further investigation and resolution to fully realize the advantages of this technology. Addressing these challenges will enable more efficient use of lcWGS for genetic research and accelerate genetic progress in cattle breeding.

## Simple Summary

Low-coverage whole-genome sequencing (lcWGS) allows reading a small portion of each animal’s DNA across the entire genome at low cost. When combined with computational methods, this approach can accurately reconstruct full genetic profiles. This review shows that lcWGS is effective for cattle genomics: it captures rare and breed-specific genetic variants better than standard SNP chips, supports accurate genomic predictions, and becomes cost-competitive. Key factors affecting its performance include sequencing depth (typically 0.5–2×), the size and composition of the reference panel, and the choice of imputation software. While challenges remain—such as optimizing protocols for crossbred animals and complex genomic regions—lcWGS is a practical and scalable tool for modern cattle breeding programs.

## 1. Introduction

Whole-genome studies in cattle play a crucial role in exploring both individual and population-level genetic variability. At the population level, such studies enable the monitoring of genetic structure, relatedness, levels of inbreeding, and loss of diversity, which is critical for the conservation of rare breeds and the rational management of genetic resources [1,2]. Moreover, they facilitate the detection of polymorphisms associated with economically important traits and their use in building accurate genomic selection models [3,4].

Genomic selection based on whole-genome genotyping data obtained from SNP arrays has become one of the key tools in modern breeding programs. However, traditional SNP chips are limited to a predefined set of variants and may not capture the full extent of genetic diversity, particularly structural variants and breed-specific sequences absent from reference genomes [5,6]. The development and updating of SNP chips also present challenges, including variant selection, probe design, and training of genotype-calling algorithms [6]. In addition, a substantial number of genomic regions may be missing from commonly used reference assemblies. For example, the incorporation of pangenome data from European and African cattle breeds revealed approximately 116 Mb of additional sequences, representing about 4% of the length of the standard reference genome [7]. This underscores the limitations of SNP chips, which rely on a reference-based framework and may fail to capture important variants.

Whole-genome sequencing (WGS) offers more accurate genomic predictions and enables the tracking of alleles associated with traits [4,8,9]. Nevertheless, despite the decreasing cost of sequencing in recent years, high-coverage WGS remains expensive, particularly when analyzing large numbers of animals, which restricts its widespread use in livestock breeding [10,11]. A promising alternative is low-coverage whole-genome sequencing (lcWGS), which provides uniform coverage of the entire genome at substantially lower cost. When combined with imputation, lcWGS enables the recovery of genotypes with high accuracy and density [6,12,13,14,15,16].

Genotypes obtained from sequencing at 0.1× coverage followed by imputation have been shown to reach accuracies comparable to those of SNP chips, particularly for rare variants, while coverage above 1× can yield substantially higher accuracy [15]. Furthermore, lcWGS provides the opportunity to identify rare and functionally important variants, including those absent from conventional SNP arrays. This is particularly valuable for the evaluation of quantitative traits and for studying breeds that are underrepresented in standard panels [4,13].

Over the past three years, significant advances have dramatically improved the practical applicability of lcWGS. Teng et al. [16] conducted a comprehensive benchmark in Holstein cattle and demonstrated that the QUILT algorithm achieves an imputation accuracy (r^2^) of 0.975 at 1× coverage, significantly outperforming Beagle v4.1, with accuracy remaining above 0.97 even at 0.5× coverage. Lamb et al. [17] demonstrated that the imputation accuracy of ONT (Oxford Nanopore Technologies) data using QUILT reached 0.98 at 2× sequencing coverage, with the highest accuracy at 0.5× coverage being 0.96. González-Recio et al. [18] confirmed that ONT with LSK114 chemistry delivers 97–99% base accuracy, enabling direct genomic values with high reliability (r^2^ = 0.79−0.99) compared to SNP arrays and while simultaneously capturing DNA methylation—a unique opportunity for epigenomic selection. Daetwyler et al. [19] showed that the specialized loimpute algorithm achieves r^2^ = 0.96−0.98 at 0.5–1× coverage. Zhang et al. [20] established the first publicly available multi-breed cattle reference panel and showed that a pipeline combining Beagle v5.4 and GLIMPSE2 yields >99.5% concordance even at 0.1× coverage in Holsteins. Most recently, novel dual-phase and deep learning–based methods like DPImpute [21] and STICI [22] have pushed the boundaries of lcWGS by enabling reliable imputation even at ultra-low coverage (<0.5×) and showing promise for structural variant reconstruction. Notably, STICI is designed as a transformer-based framework that can impute genotypes without relying on a standard reference panel, making it uniquely suited for underrepresented breeds or species where such resources are unavailable.

Currently, lcWGS is actively applied in cattle studies across both dairy and beef populations. However, several questions remain unresolved, including the choice of optimal sequencing depth, phasing and imputation tools, strategies for constructing reference panels, and the economic efficiency of large-scale implementation [23].

The aim of this study was to analyze the current scientific literature on the efficiency and potential of low-coverage whole-genome sequencing (lcWGS) as a tool for genomic research in cattle.

To date, no comprehensive review has focused specifically on lcWGS as a standalone tool for imputation-driven genomic prediction, rare variant discovery and exploratory structural variation analysis in cattle. This work aims to bridge that gap.

## 2. Materials and Methods

A literature review was conducted using the PubMed and Google Scholar databases. The following keywords and search phrases were applied in various combinations: “low-pass sequencing cattle”, “low-coverage whole genome sequencing cattle”, “genotype imputation cattle”, “genomic selection low-pass sequencing”, and “cattle WGS imputation”. The review included peer-reviewed articles in English published between 2012 and 2025. Preference was given to original research articles, systematic reviews, and large-scale projects that addressed issues related to imputation accuracy, the composition and size of reference panels, cost-effectiveness, and the practical application of lcWGS in cattle genomics.

## 3. Results

### 3.1. lcWGS as an Efficient Alternative to Traditional Genotyping Methods

Low-coverage whole-genome sequencing (lcWGS), combined with subsequent genotype imputation, represents a promising and cost-effective alternative to traditional SNP arrays for cattle genotyping [6,12,16,17,18,19,20]. In addition, lcWGS is less expensive compared to high-coverage WGS [6,24]. A key advantage of lcWGS lies in its ability to better capture rare and breed-specific genetic variants [6,18]. The demand for and efficiency of lcWGS are supported by a number of successful studies across both dairy and beef cattle.

In beef cattle, Snelling et al. [6] demonstrated that lcWGS at 1× coverage in feedlot bulls enables the generation of highly accurate imputed genotypes and molecular breeding values (MBVs), comparable in accuracy to estimates derived from SNP arrays. Similarly, Russell et al. [25] successfully applied lcWGS data imputed from ~0.5× coverage to estimate genetic parameters for growth traits in beef cattle.

In dairy cattle, Lamb et al. [17] investigated the use of Oxford Nanopore Technologies (ONT) sequencing and showed that ONT lcWGS at 0.1× coverage, followed by imputation with QUILT, yielded genomic estimated breeding values (GEBVs) with correlations exceeding 0.91 compared to SNP array-based estimates. At 0.5× coverage, ONT lcWGS outperformed low-density SNP arrays in both genotyping accuracy and GEBV correlations. Furthermore, González-Recio et al. [18] demonstrated the effectiveness of ONT lcWGS (using LSK114 chemistry at 2× coverage) for obtaining direct genomic values (DGVs) in dairy cattle, achieving high reliability (r^2^ = 0.79−0.99 compared to SNP arrays).

These examples, along with the studies focusing on the optimization of imputation protocols [12,16,19,20], highlight that lcWGS is a practical and valuable tool for modern genomic research and breeding programs in cattle.

### 3.2. Genotype Imputation from lcWGS Data

The efficiency of lcWGS applications directly depends on the accuracy with which complete genotypes can be imputed from low-coverage sequencing data. Modern imputation algorithms, when combined with high-quality reference panels, enable highly accurate genotype reconstruction. Depending on sequencing depth, software tools, and reference panel characteristics, accuracy metrics such as concordance (the proportion of correctly imputed genotypes) and the squared correlation coefficient (r^2^) often exceed 0.95 [12,16,20].

For instance, Zhang et al. [20], using a multi-breed reference panel of 2976 animals and a combination of Beagle v5.4 and GLIMPSE2, achieved concordance rates of 99.6% at 0.5× and 1× coverage, and 99.5% even at 0.1× coverage. Snelling et al. [6] reported that genotypes imputed from 1× lcWGS in beef cattle showed a very high correlation (r = 0.99) with SNP array-derived genotypes. Using Oxford Nanopore Technology (ONT), Lamb et al. [17] demonstrated that genotype imputation accuracy at 0.5× coverage surpassed that of low-density SNP arrays. Similarly, Daetwyler et al. [19] reported high imputation accuracy with the software loimpute (https://gitlab.com/gencove/loimpute-public, accessed on 2 December 2025), with correlations of 0.92 at 0.5× and 0.93 at 1× coverage.

These findings indicate that even at very low sequencing depths, modern lcWGS and imputation approaches can provide genomic data of high quality.

Imputation accuracy (Table 1) is influenced by a complex set of factors: the algorithm used, the size and composition of the reference panel, the degree of relatedness between the panel and the target sample, minor allele frequency (MAF), and the quality of the input data [8,26,27,28].

Sequencing depth is a key factor. Accuracy increases with higher coverage, as a greater number of reads provides more reliable information for haplotype reconstruction [12,16]. According to Lloret-Villas et al. [12], the F1 imputation metric rose substantially when coverage increased from 0.01× to 1×, with a less pronounced gain up to 4×. Teng et al. [16] reported a similar trend for most tested algorithms. González-Recio et al. [18] found that coverage of ~2× is needed to achieve direct genomic values (DGV) with minimal bias, although high rank correlations can already be obtained at 0.5×. Thus, optimal coverage is defined by a balance between accuracy and cost. In most practical cases, 0.25×–1× is sufficient, particularly when modern algorithms and robust reference panels are used [17,20].

The impact of coverage depth can be modulated by other factors, such as reference panel quality. Higher coverage can partly compensate for panel shortcomings [12]. The quality, size, and composition of the panel exert a decisive effect on accuracy [24]. Increasing the number of animals in the reference panel improves imputation accuracy, especially for low-MAF variants [2,12,20,30].

The composition of the animal reference panel—single-breed or multi-breed—is also important. For within-breed imputation, sufficiently large single-breed reference panels often provide the highest or at least comparable accuracy relative to multi-breed panels of the same size [12,28]. This is because haplotypes within a breed are more conserved. Multi-breed panels may be useful to increase reference sample size, especially when single-breed resources are limited [1,20]. However, adding genetically distant breeds or too many unrelated animals to an already representative panel may be unhelpful or even detrimental, introducing “noise” or irrelevant haplotypes [12,28,31]. It is critical that multi-breed panels include sufficient representation of the target breed or closely related breeds [1,31]. Imputation in crossbred animals is more challenging due to mixed linkage disequilibrium structure, resulting in lower accuracy compared with purebreds [31,32]. Specific strategies may be needed, such as panels including parental breeds and crossbreds themselves [1,31]. Improved phasing algorithms also enhance imputation accuracy [17].

MAF is one of the key factors determining imputation accuracy. Accuracy increases with higher MAF. The effect is most evident for rare variants with MAF < 0.05 [16]. Lloret-Villas et al. [12] noted that increasing coverage from 0.25× to 1× markedly improved accuracy for MAF < 0.05. GLIMPSE and QUILT are less affected by MAF and show greater efficiency for rare variants compared with Beagle, GeneImp, and IMPUTE5 [16,17].

The choice of software and underlying algorithms for haplotype phasing and imputation is another critical factor (Table 2). Programs specifically designed or adapted for lcWGS often perform better. These include QUILT, GLIMPSE, and commercial tools such as Gencove’s loimpute [16,17,19]. Beagle is widely used for both phasing and imputation. Combining Beagle for phasing with GLIMPSE2 for imputation has been recommended as a fast and accurate pipeline for lcWGS in cattle [20]. STITCH, which can operate without an animal reference panel, is useful when reference resources are limited; subsequent imputation with Beagle can increase the number of imputed SNPs [16]. Multiple studies have compared software performance. Teng et al. [16] showed that GLIMPSE, QUILT, and STITCH generally outperformed Beagle v4.1 and GeneImp. Daetwyler et al. [19] reported significant advantages of loimpute over Beagle 4.0. Earlier SNP-chip imputation studies also highlighted differences: FImpute was often faster and more accurate than Beagle 3.3.2 and Impute2, particularly for low-density panels and when pedigree information was available [1,32]. Proper parameter tuning is important. For example, the effective population size (Ne) parameter in Beagle should be adapted for livestock populations, as default values optimized for humans may reduce accuracy [2]. Modern versions, such as Beagle 5.x and IMPUTE5, have been substantially optimized for speed and memory usage, enabling work with very large reference panels [2,33]. Deep learning–based methods (e.g., transformer architecture in STICI) are emerging, promising improved efficiency and accuracy, especially for complex variant types (structural, multiallelic), and may reduce dependence on traditional reference panels [22]. Online platforms are also becoming available, such as AGIDB, a database of various livestock species providing genotype imputation and variant annotation interfaces. It integrates WGS and SNP-chip data across multiple species, including cattle, and offers ready-to-use references, analytics, and visualization, making it highly useful for lcWGS imputation and livestock genomics research [34]. Zheng et al. [21] developed DPImpute (Dual-Phase Impute), a two-round imputation system using GLIMPSE and IMPUTE2. DPImpute addresses the challenge of accurately imputing tens of millions of SNPs at sequencing depths below 0.5×, showing superior accuracy compared to existing systems when using limited test samples (≤10) and reference samples (≤100). For broader adoption, a web server with a graphical interface has been developed.

**Figure 1:**
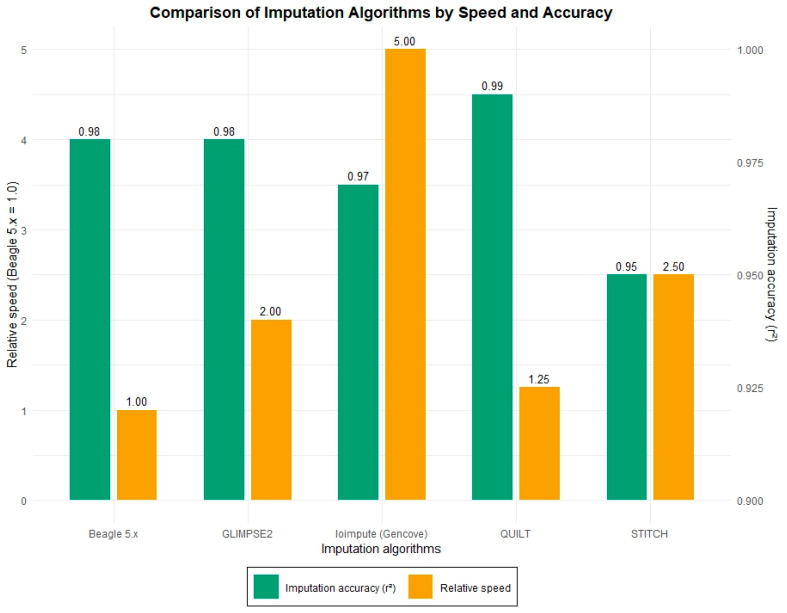
Comparison of popular lcWGS imputation algorithms by accuracy (r^2^) and relative computational speed. Note. Data are based on Teng et al. [16], Zhang et al. [20], Jiang et al. [2], Daetwyler et al. [19], and Snelling et al. [6]. Speed is normalized relative to Beagle 5.x (value 1.0). Accuracy (r^2^) is shown under typical lcWGS conditions (coverage ~1×, reference panel size >1000 animals). Modern tools enable imputation with accuracy exceeding 0.95. Regarding speed, specialized commercial software generally outperforms other programs.

Figure 1 shows a comparison of popular algorithms by two key parameters, r^2^ and conditional speed: QUILT, GLIMPSE2, Beagle5.2, STITCH, and loimpute.

Modern software tools allow genome imputation with accuracy above 0.95. In terms of speed, specialized commercial software outperforms other programs.

### 3.3. Ability of lcWGS to Detect Genetic Variability

One of the fundamental advantages of low-coverage whole-genome sequencing (lcWGS) over traditional SNP chips is its ability to provide broader coverage of genetic variation in the studied populations, including detection of variants absent from chips and identification of more complex forms of variation (deletions, insertions, CNVs, etc.). Standard SNP chips contain a fixed set of polymorphisms, often biased toward common variants, which limits the detection of rare or breed-specific alleles, especially when such breeds were not included in chip design [6,18]. This limitation can lead to loss of important genetic information associated with quantitative traits, adaptation, or genetic disorders, and complicates accurate estimation of genetic diversity and inbreeding, particularly in small or local breeds. Since lcWGS provides coverage of the entire genome rather than fixed sites, it forms a basis for genotype imputation across the full spectrum of allele variants, including rare ones present in the reference panel [24]. Snelling et al. [6] noted that lcWGS enables imputation of genotypes for tens of millions of variants, including a large number of potentially functional (protein-altering and regulatory) variants absent from SNP chips. González-Recio et al. [18] also emphasized that lcWGS can be effective for populations requiring high marker density across a broad allele frequency range, including rare variants. Although imputation accuracy for rare variants may be lower than for common ones [16,19], specialized imputation methods and large reference panels improve accuracy [2,17]. The ability of lcWGS to capture and support imputation of such variants is critical for a deeper understanding of the genetic architecture of quantitative traits, accurate monitoring of genetic diversity, and development of effective genetic resource conservation strategies.

Despite theoretical potential, the accurate detection and imputation of structural variants (SVs) from typical low-coverage WGS (0.1–2×) remains a major challenge. Recent protocols demonstrate that reliable SV calling is feasible at 4–10× coverage only when multiple complementary algorithms (e.g., Manta, Lumpy, SVseq2) are combined and stringent post-filtering is applied to exclude variants overlapping repetitive or low-complexity regions [41]. Furthermore, genotyping SVs benefits significantly from graph-based reference panels (e.g., via Graphtyper2), which represent complex genomic architectures better than linear references. At the ultra-low coverages (<2×) commonly used in large-scale cattle genomics, SV analysis is currently impractical without such specialized pipelines.

While most lcWGS studies focus on SNPs and small indels, recent work demonstrates its potential for structural variant (SV) detection. Zan et al. [42] used ~0.5–1× lcWGS in a chicken F2 cross to identify large (>10 kb) deletions associated with body weight and behavior, validated by qPCR and Sanger sequencing. Their method (Stripes) leverages read-depth anomalies to genotype SVs directly from lcWGS data, without requiring high-coverage WGS. Critically, these SVs explained up to 50% of phenotypic variance—far more than surrounding SNPs—highlighting their functional importance. Although hybrid designs simplify phasing, tools like STICI [22] and DPImpute [21] now extend SV imputation to outbred livestock populations using graph-based reference panels. This suggests that lcWGS, when combined with specialized pipelines, can unlock the “hidden heritability” residing in structural variation.

lcWGS generates reads across the whole genome, which after imputation allows the reconstruction of genotypes at much higher density, approaching that of high-coverage WGS [6]. This provides a more detailed view of haplotype structure and linkage disequilibrium (LD) across the genome. Standard reference genomes may be incomplete or fail to reflect the full diversity of genomic sequences across cattle breeds. Pan-genome studies have shown that significant genomic regions may be absent from standard reference genomes [7]. lcWGS creates opportunities for analyzing not only SNPs but also other types of genetic variation, such as small insertions/deletions (indels) and larger structural variants (SVs). Even though SV imputation at low coverage remains technically challenging, ongoing progress in graph-based approaches and AI-assisted imputation frameworks is expected to improve its reliability, making lcWGS a viable foundation for comprehensive genomic profiling in cattle populations.

### 3.4. Application of lcWGS in Genomic Selection

Implementation of low-coverage whole-genome sequencing (lcWGS) opens new opportunities to improve the efficiency of genomic selection programs in cattle. This is achieved through more accurate genomic estimated breeding values (GEBV), due to broader coverage of genetic variation, and through potential reduction in genotyping costs. Several studies have compared the accuracy of GEBV based on lcWGS-imputed genotypes with those derived from traditional SNP chips or high-coverage WGS. Snelling et al. [6] showed in beef cattle that molecular breeding values (MBV) predicted using lcWGS genotypes at 1× coverage were highly consistent with MBV obtained from SNP chips (BovineSNP50, GGP-F250). Correlations between MBV from lcWGS and SNP chips exceeded 0.96. Correlations with traditional EBV and GEBV were also similar for both sources, demonstrating lcWGS as a reliable genomic prediction base. Lamb et al. [17] reported that when using QUILT for imputation, GEBV for beef traits from lcWGS ONT data at only 0.1× coverage showed high correlation (>0.91) with SNP chip–based high-density GEBV. At 0.5× coverage, lcWGS-based GEBV surpassed low-density chip results. González-Recio et al. [18] found in dairy cattle that direct genomic values (DGV) from lcWGS ONT (latest LSK114 chemistry, 2× coverage) were highly accurate (r^2^ = 0.79−0.99) compared with chip-based DGV. VanRaden et al. [4] also observed that WGS yields more accurate genomic predictions. lcWGS can enhance genomic selection programs through:

Broader causal variant coverage: Unlike SNP chips limited to LD markers, lcWGS + imputation provides data on many more variants, including causal mutations or variants in stronger LD [6,24]. This is especially valuable for multi-breed populations where LD patterns differ [1].

Inclusion of rare variants: Many economically important traits and genetic defects are controlled by rare variants not represented on chips. lcWGS enables their detection and imputation, improving prediction accuracy and aiding selection against deleterious alleles [4,13].

Support for resource-limited breeds: Developing breed-specific chips is often impractical. lcWGS provides a flexible, cost-effective alternative, especially with panels including related breeds or international consortium data [18,20].

Lower costs, broader coverage: Declining sequencing costs make lcWGS competitive with SNP chips while allowing genotyping of larger populations [17,19]. Larger reference populations improve prediction accuracy [6].

Marker flexibility: lcWGS provides millions of sites after imputation, allowing dynamic marker subsets for analyses and model updates without switching platforms [6,25].

Integration with epigenomic data: ONT-based lcWGS can also capture DNA methylation, enabling future selection models that include epigenetic effects [18].

Thus, lcWGS has strong potential to transform genomic selection in cattle, offering accurate, flexible, and cost-effective genotyping and prediction strategies.

The technological benefits of lcWGS ultimately need to be validated through real breeding applications, which are explored in the next section.

### 3.5. Economic Efficiency of lcWGS

In addition to its technological advantages, economic efficiency is a key factor determining the prospects for wide adoption of low-coverage whole-genome sequencing (lcWGS) in cattle genomics and breeding. The main advantage of lcWGS is the ability to obtain high-density genomic data at much lower cost than high-coverage WGS, and often at costs comparable to or lower than medium- and high-density commercial SNP chips. High-coverage WGS provides the most complete genetic information but remains too expensive for routine large-scale genotyping [10]. lcWGS requires far less sequencing per sample (0.1×–2×), which reduces primary data costs while still allowing accurate imputation across the genome [6,12].

Several studies indicate that lcWGS is already cost-competitive with SNP chips. Snelling et al. [6] noted that falling sequencing costs and advances in high-multiplex library preparation make lcWGS competitive with chips. Daetwyler et al. [19] reported that lcWGS genotyping is comparable in price to low- and medium-density chips while providing WGS-equivalent data density. Lamb et al. [17] showed that with proper multiplexing (up to 40 human-sized genomes per MinION flow cell), ONT-based lcWGS reagent costs can drop below $40 per sample. González-Recio et al. [18] emphasized that ONT lcWGS can also be cost-effective, especially given its ability to simultaneously deliver epigenetic information without added cost.

Economic evaluation must account not only for sequencing but also for library preparation, bioinformatics, and imputation. Even with these costs, lcWGS often remains attractive [23]. It also eliminates expenses linked to chip design, manufacturing, and updating [6]. Cost-effectiveness depends on research goals, genotyping scale, and available alternatives. lcWGS is particularly economical for large-scale studies, where multiplexing greatly reduces per-sample costs [17]. This is crucial for genomic selection programs requiring thousands of animals in reference populations and evaluations of young stock.

For cases requiring rare, breed-specific, or chip-absent variants, lcWGS is more cost-effective than custom chip development or sequencing all samples at high coverage [6,18]. For breeds lacking commercial high-density chips, lcWGS is the only affordable way to obtain genome-wide data [18]. Additional information such as DNA methylation, available via ONT, further increases lcWGS value without substantial cost [18]. Sequencing data can be reanalyzed as reference genomes and methods improve [6,25].

Overall efficiency depends on optimizing protocols, including minimal sufficient coverage, fast imputation algorithms, and reference panel strategies [23].

lcWGS is an economically attractive genotyping strategy for cattle, balancing cost, genomic information, and quality. With declining sequencing costs and improved bioinformatics, lcWGS is becoming increasingly practical for large-scale livestock applications.

### 3.6. Challenges, Limitations, and Open Questions

Despite major progress and strong potential of low-coverage whole-genome sequencing (lcWGS) in cattle genomics, several challenges, limitations, and open questions remain before its benefits can be fully utilized. Open questions include:

Technical level (coverage, imputation algorithms). Multiple studies show that very low coverages (0.1×–0.5×) can work under certain conditions [17,20], but the optimal lcWGS depth depends on study goals (e.g., genomic selection for polygenic traits, discovery of rare causal variants, population structure), sequencing technology (short vs. long reads), reference panel quality and size, and population genetics [12,23]. For precise estimation of direct genomic values (DGV) with the latest ONT chemistry, ~2× coverage may be required, whereas lower coverage can suffice for rank correlations [18]. More research is needed to define economically and statistically adequate depths for different lcWGS use cases in cattle. Input data quality and platform-specific errors: lcWGS and imputation accuracy depend on DNA quality and sequencing platform errors. ONT accuracy, while improving, can be lower than Illumina [18]. Sample issues such as twin DNA chimerism can also cause errors [6].

Resource level (reference panels). Reference panel quality, size, and composition are critical for imputation accuracy [12,24]. Panel threshold sizes need to be determined for acceptable accuracy across breeds and lcWGS depths, balancing accuracy with costs of building and maintaining panels [33]. Comparing within-breed and multi-breed panel compositions, while within-breed panels often yield high accuracy [12,28], building them for all breeds is difficult. Multi-breed strategies, breed inclusion criteria, and genetic distance must be optimized [1,20].

Application level (crossbred cattle, complex variations). Imputation for crossbreds and specific genetic groups allows many chances for errors. Achieving high accuracy in crossbreds and in breeds with distinctive backgrounds (e.g., Bos indicus) remains challenging and requires tailored panels or adaptations [6,31,32]. Imputing complex variant types and regions requires special proficiency. Current lcWGS strategies work well for biallelic SNPs, but accurate imputation of SVs, multiallelic variants, and difficult regions (high GC, segmental duplications) is still a major challenge [20,22]. Methods that handle these from lcWGS are needed. Epigenomic integration is another source of problems. While ONT-based lcWGS can yield methylation data [18], questions remain about reliability at low overall coverage and how best to integrate these data into genomic evaluation and selection models.

Standardization level (processes, verification). The standardization of pipelines and the choice of software are crucial. Many tools exist for alignment, variant calling (if used pre-imputation), phasing, and imputation [16,19,20]. Performance varies with read type, coverage, panel size, and population [16,17,19]. Default human-oriented parameters (e.g., Beagle’s Ne) often need livestock-specific tuning [2]. Lack of standard pipelines hinders comparability and adoption [23]. Assessing the downstream impact of imputation errors can be detrimental to results. Even with high mean accuracy, errors occur, especially for rare variants or complex regions [20,24]. Their effects on GWAS, diversity metrics, and GEBV accuracy/bias must be quantified. Metrics should capture not only average accuracy but also per-variant or regional reliability [18]. Broader validation. Many lcWGS studies involve limited breeds or contexts. Wider validation across dairy, beef, composite, and local breeds, varied breeding systems, and diverse traits is needed to confirm general applicability.

Addressing these issues will enable lcWGS to deliver maximum value for genetic research and to accelerate genetic progress in cattle breeding. While the Results section summarizes empirical findings, the following Discussion aims to interpret these outcomes in the context of genetic mechanisms and previous research.

## 4. Discussion

Low-coverage whole-genome sequencing (lcWGS) is transforming into a practical genotyping tool for cattle genomics. Our review synthesizes current evidence demonstrating that lcWGS combined with imputation represents a transformative technology that expands the scope and resolution of genetic analyses in cattle populations.

### 4.1. Principles and Technological Background of lcWGS

While SNP chips analyze a fixed, pre-selected set of variants, lcWGS provides sparse (0.1–2×) but uniform coverage of the entire genome. The power of this approach is realized through imputation, which utilizes haplotype reference panels derived from high-density WGS to reconstruct complete individual genotypes with high accuracy [1,12].

The lcWGS process integrates three key components: the sequencing platform (short-read such as Illumina or long-read such as Oxford Nanopore), phasing algorithms (e.g., Beagle), and imputation algorithms (e.g., GLIMPSE2, QUILT). In lcWGS applications, Illumina sequencing typically produces a base error rate of ~0.1–1%, while ONT platforms historically reached ≥5–10% but have recently decreased to 1–3% with LSK114 chemistry, making long-read lcWGS a sufficiently effective option that additionally enables methylation analysis, a feature unavailable with chips [18]. Such improvements allow ONT to deliver additional epigenetic information, although at very low coverage (<1×), Illumina tends to ensure higher imputation reliability.

The primary advantage of lcWGS is its ability to capture a broader spectrum of genetic variation. It facilitates the imputation of tens of millions of variants, including rare (MAF < 0.05), breed-specific, and potentially functional alleles that are systematically absent from standard SNP chips [6,18]. This provides a more comprehensive view of genomic architecture, which is crucial for detailed genetic studies and accurate genomic predictions.

While the technological principles underpinning lcWGS explain its theoretical advantages, a comprehensive assessment requires evaluating how these benefits translate into practical applications across breeding, population management, and genomic research.

A sequencing depth of 0.5–1× is generally optimal for routine genomic prediction, whereas studies targeting rare variant discovery or epigenetic profiling may benefit from increasing coverage to ≥2×. Zhu et al. [43] demonstrated in a population of 3579 Duroc pigs that lcWGS at ~0.7× coverage, followed by imputation using a reference panel of 100 high-coverage genomes, yielded 3–5% higher genomic prediction accuracy compared to the commercial 50K SNP chip—particularly for low-heritability traits. MacLeod et al. [44] showed that imputation from lcWGS data at 0.4× coverage achieved a genome-wide r^2^ of 0.93, significantly higher than the r^2^ of 0.83 obtained from 50K SNP chip imputation. This advantage was especially pronounced for rare variants (MAF < 0.01), which are frequently missed in SNP chip design but may underlie important functional effects. Consequently, genomic prediction accuracy improved by 2–4% for production and reproductive traits.

### 4.2. Applications Beyond Genomic Selection (The Detection of Recessive Disorders, Analysis of Rare Variants, Assessment of Genetic Diversity, and Use in GWAS or Population Structure Studies)

Although genomic selection remains the primary use case for lcWGS, its utility extends far beyond breeding value estimation. lcWGS enables genome-wide screening for deleterious recessive alleles in heterozygous carriers and identifies causal mutations absent from standard chips.

A promising application of lcWGS is the investigation of structural variations (SVs)—deletions, duplications, insertions, and inversions. While SNP chips largely “miss” SVs, the uniform genome-wide coverage of lcWGS provides a basis for their detection. SVs can have a profound impact on gene function, adaptation, and complex traits. For instance, mapping SVs in populations can reveal variants associated with local adaptation, disease resistance, or production traits that would be missed by SNP-based approaches [41]. This makes lcWGS a powerful tool for studying the genetic architecture of important phenotypes influenced by large genomic rearrangements. While lcWGS enables preliminary SV detection, accurate imputation of large indels or CNVs from short-read low-coverage data remains limited. Recent advances using deep learning (e.g., STICI) and long-read integration (ONT) demonstrate promising improvements [17,22].

Standard metrics such as runs of homozygosity (ROH) and genomic inbreeding coefficients (FROH) are more accurately estimated from the dense genome-wide data obtained from imputed lcWGS genotypes compared to sparse chip data. This is particularly important for conservation programs and monitoring genetic resources of rare and local breeds [1,7,45]. lcWGS enables reliable estimation of allele frequencies, genetic diversity (π, θ), inbreeding (FROH), and population structure—even at coverage as low as 0.1–0.5×, provided that the sample size is sufficiently large [46]. It has been demonstrated that lcWGS with an average coverage of just 1.4× can identify five genetically distinct breeding populations in the American Redstart (Setophaga ruticilla)—a species exhibiting extremely low genome-wide differentiation (mean FST = 0.009) [47]. A powerful and cost-effective application is individual identification and paternity testing. Simulation studies have shown that ultra-low-coverage lcWGS (as low as 0.05×) can achieve >99% accuracy for both traceability and paternity testing [48]. This allows the same sequencing data to serve a dual purpose: fulfilling legal pedigree requirements and providing input for genomic evaluation, thereby eliminating redundant genotyping costs [49].

The high marker density from lcWGS improves the resolution of genome-wide association studies (GWAS), enabling more precise mapping of causal regions. It also enhances the accuracy of population structure analysis in admixed or crossbred populations, where chip-based markers may be uninformative due to differing LD patterns [4,31].

Work by Erven et al. [45] demonstrated that imputation from lcWGS (0.25×) allows for accurate reconstruction (>99.1%) of genotypes from a Mesolithic aurochs (~9800 BP), opening avenues for reconstructing domestication history and demographic dynamics of ancient populations.

The utility of lcWGS extends to functional genomics. Widmayer et al. [50] demonstrated in the genetically diverse Diversity Outbred mouse model that lcWGS at ~0.9× coverage enables not only highly accurate genotype imputation but also precise haplotype reconstruction and expression quantitative trait locus (eQTL) mapping. Using the QUILT imputation pipeline, they identified millions of variants—far exceeding the yield of ddRAD-seq—and successfully linked genotypes to transcriptomic variation across 183 cell lines. This illustrates that lcWGS data can serve as a foundation for systems genetics, facilitating the discovery of causal genes and regulatory mechanisms underlying complex traits.

Given that the reliability of lcWGS-based analyses largely depends on algorithms and reference data, it is crucial to consider recent technological developments that have improved imputation accuracy and analytical scalability.

### 4.3. Recent Technological and Bioinformatic Advances

The impressive performance of lcWGS is driven by continuous innovation in computational methods and the expansion of reference data.

Chat et al. [51] demonstrated that lcWGS at 0.4× coverage, combined with modern imputation (GLIMPSE), outperforms SNP arrays in both variant recovery and genotype accuracy. The development of specialized tools such as QUILT, GLIMPSE2, and the commercial loimpute has been crucial. These algorithms enable imputation accuracy (r^2^) exceeding 0.97 even at 0.5× coverage in cattle [16,17]. New methods like DPImpute (a dual-phase framework) and STICI (a transformer-based deep learning model) promise further advances, especially for ultra-low coverage (<0.5×) and for complex variant types like SVs and multiallelic sites [21,22].

Protocols have been developed that combine multiple complementary algorithms (e.g., Manta, Lumpy, SVseq2) for reliable detection of SVs from low-coverage data (typically 4–10×), utilizing various signals such as read pairs, split reads, and read depth [41]. Subsequent population-scale genotyping of SVs can be performed using graph-based tools like Graphtyper2, which uses pangenome-aware panels for accurate genotyping. This integrated approach enables the construction of population-level SV maps from lcWGS data.

The size and diversity of reference panels are crucial. Large-scale projects like the “1000 Bull Genomes Project” (including >6000 animals [52] of various breeds) have significantly improved imputation accuracy within populations [20]. Most current lcWGS studies are based on European and North American breeds; validation on African, Asian and locally adapted composite populations is still limited and requires further investigation before broad implementation. In such cases, algorithms that do not require reference panels, such as STITCH [16], can be used.

The emergence of user-friendly resources like the AGIDB database, which provides imputation and variant annotation capabilities, is making bioinformatics accessible to a wider range of researchers and breeders [34].

Considering these technological developments and current limitations, it is necessary to outline how lcWGS may evolve and how future research and infrastructure can drive its adoption in livestock breeding.

### 4.4. Future Perspectives

Looking ahead, lcWGS is likely to become a standard genotyping tool in cattle breeding programs. The ongoing development of cattle pangenome references [7] will shift lcWGS imputation from linear-reference alignment to graph-based mapping. This will help address current limitations in SV analysis.

The ability of ONT-based lcWGS to provide methylation profiles at no extra cost opens the door for “epigenomic estimated breeding values” that could account for plasticity in response to environmental factors [18].

For large-scale breeding programs (>200 animals), lcWGS becomes cost-competitive with SNP arrays. However, for small-scale operations (<30 animals), chip-based genotyping may remain more practical. As sequencing costs continue to decline, lcWGS will become increasingly cost-competitive. The current cost of 0.5× short-read sequencing (typically 8–12 USD/sample) followed by imputation is comparable or lower than high-density SNP chips (35–45 USD), particularly in large-scale breeding schemes [19,20].

Thallman et al. [53] propose a transformative framework that addresses both challenges. They introduce a haplotype-based compression system in which an individual’s genome is represented as an array of identifiers referencing pre-defined haplotypes across approximately 50,000 genomic segments. This approach reduces storage requirements to ~200 KB per animal—comparable to a high-density SNP chip—while preserving full sequence resolution.

Despite progress, challenges remain. Achieving high imputation accuracy in crossbred animals and for complex genomic regions requires further methodological refinement and more diverse reference panels. This implies demonstrating not only technical superiority but also consistent, economically significant improvements in breeding outcomes. This can be achieved not by brute-force inclusion of all variants, but through strategic, functionally informed filtering of imputed data.

In conclusion, addressing these challenges through collaborative efforts to build diverse references, standardize pipelines, and validate economic benefits will ensure that lcWGS fully realizes its potential as a scalable, accurate tool, thereby accelerating genetic progress and promoting sustainable cattle breeding worldwide.

## 5. Conclusions

Low-coverage whole-genome sequencing (lcWGS) has proven to be a viable and increasingly cost-effective alternative to SNP arrays for cattle genotyping. With sequencing depths of 0.5–2× and appropriate imputation pipelines, it achieves high genotype accuracy (>0.95) and enables the detection of rare, structural, and breed-specific variants that are often missed by commercial chips. Its utility in genomic selection has been demonstrated across both dairy and beef populations, with genomic predictions comparable to or better than those based on SNP arrays.

The performance of lcWGS depends critically on three factors: (1) sequencing depth, where 0.5–1× is often sufficient for routine applications; (2) reference panel size and composition, with within-breed panels generally outperforming multi-breed ones for purebred animals; and (3) the choice of imputation software, with tools like GLIMPSE, QUILT, and loimpute showing superior accuracy for low-coverage data.

Remaining challenges include improving imputation for crossbred animals, rare variants, and complex genomic regions, as well as standardizing bioinformatics pipelines. Nevertheless, with ongoing advances in sequencing technology, reference resources, and computational methods, lcWGS is well positioned to become a standard tool in cattle genomics and breeding programs worldwide.

## Figures and Tables

**Table 1:** Accuracy of genotype imputation from lcWGS in different cattle studies.

Sequencing Depth	Sample Size /Breed	Imputation Software	Reference Panel (Animals)	Applicable Scenarios	Accuracy Metric	Reported Accuracy	Source
1×	n = 77 crossbred beef cattle	loimpute	946 (multi-breed)	Genomic prediction in crossbred beef cattle using a multi-breed reference panel	r (vs. HD SNP array)	0.99	[6]
0.5×, 1×	n = 31 Holstein; n = 55 Jersey; n = 39 Holstein × Jersey crossbred bulls	loimpute, Beagle v5.1	4109 (incl. 1200 Holstein, 120 Jersey, 1000 Bull Genomes Run 8); Gencove reference panel—946 animals (incl. 184 Holstein, 15 Jersey)	Genomic selection in dairy cattle and their crosses using large, breed-representative reference panels	Concordance	loimpute: 0.96–0.98 (0.5×), 0.95–0.96 (1×); Beagle: 0.87–0.89 (0.5×), 0.91–0.92 (1×)	[19]
0.1×–1×	n = 62 Holstein; n = 66 Simmental	Beagle v5.4 + GLIMPSE2	2976 (multi-breed)	Large-scale genomic prediction in mainstream dairy and beef breeds using a public multi-breed reference panel	Concordance	Holstein: 99.6% (1×), 99.6% (0.5×), 99.5% (0.1×); Simmental: 98.8% (1×)	[20]
0.25×	n = 24 Brown Swiss	GLIMPSE v1.1.1	150 (multi-breed)	Cost-effective genotyping for population studies in minor or underrepresented breeds when large within-breed panels are unavailable	F1-score	>0.9	[12]
1×	n = 800 Holstein	Beagle v4.1, GeneImp v1.3, GLIMPSE v1.1.0, QUILT v1.0.0, Reveel, STITCH v1.6.5	1059 (1000 Bull Genomes Project Run 8)	Benchmarking and high-accuracy genomic prediction in intensively selected purebred populations with large breed-specific reference panels	r^2^	Beagle: 0.94; GeneImp: 0.95; GLIMPSE: 0.96; QUILT: 0.97; Reveel: 0.53; STITCH_REF: 0.98; STITCH: 0.98	[16]

Note. The r^2^ is the squared correlation between the expected dosages (posterior expectation of the imputed allele dosages) and the known true genotypes [29]. The concordance rate, also known as the accuracy ratio, is the ratio of the number of samples in the imputed dosage that are correct compared to the total number of samples in the true dosage [20]. F1 scores—harmonic mean of precision and recall [12].

**Table 2:** Comparison of popular lcWGS imputation software in cattle.

Software	Advantages (Based on Articles)	Features/Considerations (According to Literature)	Applicable Scenarios	Sources
Beagle v.4.1, v.5.1, v.5.4 [35]	Widely used; high accuracy with correct Ne settings	Can be slower than specialized tools; Ne tuning is crucial for cattle	Legacy genomic prediction pipelines using SNP-array-based imputation or when high-quality called genotypes (not raw BAMs) are available for lcWGS data	[2,12,20]
GLIMPSE v1.1.0/GLIMPSE2 [36]	Good accuracy, works with genotype probabilities, robust to MAF	Can be slower than Beagle for phasing (unless used in combination)	Large-scale lcWGS studies (0.1–1×) in purebred or well-represented breeds with sufficiently large (≥75–150 animals) within-breed reference panels	[16,17,20]
QUILT v1.0.0, v1.0.1 [37]	Very high accuracy for lcWGS (especially ONT)	Can be slow with high coverage and large SNP reference panels	High-accuracy imputation for research applications (e.g., rare variant discovery) when a large (≥1000), breed-specific reference panel is available	[16,17]
loimpute (Gencove) [38]	Specialized for lcWGS, high accuracy, robust to MAF	Commercial software (Gencove)	Practical implementation in breeding programs using low-coverage data (0.25–1×), particularly for cross-bred cattle	[6,19]
STITCH v1.6.5 [39]	Can operate without a reference panel of animals	Fewer imputed SNPs; accuracy highly dependent on coverage depth and number of samples	Reference-free imputation in large cohorts (≥400 animals, ≥0.4× coverage) when no suitable reference panel is available	[16]
STICI [40]	Potential for SVs and multi-allelic variants; no traditional reference panel required	New method; requires validation for lcWGS in cattle	Experimental applications, particularly for structural variation detection and analysis in crossbred or less-characterized breeds	[22]

## Data Availability

All the data is available in the article.
